# New era for mucosal mast cells: their roles in inflammation, allergic immune responses and adjuvant development

**DOI:** 10.1038/emm.2014.7

**Published:** 2014-03-14

**Authors:** Yosuke Kurashima, Hiroshi Kiyono

**Affiliations:** 1 Division of Mucosal Immunology, Department of Microbiology and Immunology, Institute of Medical Science, University of Tokyo, Tokyo, Japan; 2 Core Research for Evolutional Science and Technology (CREST), Japan Science and Technology Agency (JST), Tokyo, Japan; 3 Division of Infectious Genetics, Department of Microbiology and Immunology, Institute of Medical Science, University of Tokyo, Tokyo, Japan; 4 Department of Medical Genome Science, Graduate School of Frontier Science, University of Tokyo, Chiba, Japan; 5 International Research and Development Center for Mucosal Vaccines, Institute of Medical Science, University of Tokyo, Tokyo, Japan

**Keywords:** food allergy, intestinal inflammation, mucosal mast cells

## Abstract

To achieve immune homeostasis in such a harsh environment as the intestinal mucosa, both active and quiescent immunity operate simultaneously. Disruption of gut immune homeostasis leads to the development of intestinal immune diseases such as colitis and food allergies. Among various intestinal innate immune cells, mast cells (MCs) play critical roles in protective immunity against pathogenic microorganisms, especially at mucosal sites. This suggests the potential for a novel MC-targeting type of vaccine adjuvant. Dysregulated activation of MCs also results in inflammatory responses in mucosal compartments. The regulation of this yin and yang function of MCs remains to be elucidated. In this review, we focus on the roles of mucosal MCs in the regulation of intestinal allergic reaction, inflammation and their potential as a new target for the development of mucosal adjuvants.

## Introduction

Cutaneous and mucosal compartments are continuously and directly exposed to outside environments and are the major pathogen invasion sites. The physical barrier of the epithelial layer, mucin and antimicrobial peptides protect these tissues.^1^ Various host cells are involved in innate and acquired immunity, including innate lymphoid cells, dendritic cells (DCs) and T cells in the surface compartments. Even though these cells are generally critical for the induction of protective immunity, some of them are occasionally abnormally activated by nonpathogenic stimuli such as food allergens (for example, eggs or wheat) or endogenous ligands (for example, heat-shock proteins or uric acid), and consequently cause allergic or inflammatory diseases.^2, 3^ Among various innate immune cells, mast cells (MCs) are involved in various immunological homeostases and disorders.^4, 5^

MCs possess the high-affinity IgE receptor Fc epsilon receptor (FcɛR) on their surface; crosslinking of this receptor by an immunocomplex of the allergen-specific IgE and the allergen induces degranulation and release of histamine and effector proteases (for example, tryptase and chymase), leading to the development of allergic diseases.^6, 7^ MCs are divided into two types on the basis of protease expression patterns.^8^ In mice, mucosal-type MCs, located in mucosal compartments, express chymase (mouse mast cell protease (mMCP)-1 and mMCP-2).^8^ Connective tissue-type MCs, located in the skin and blood vessels, express chymase (for example, mMCP-4, mMCP-5), tryptase (mMCP-6 and mMCP-7) and carboxypeptidase A.^8^ Both MC types are involved in allergic reactions: mucosal MCs in the intestinal mucosa and connective tissue MCs in the skin or in systemic allergies.^8^ Proteases produced by MCs have been detected in the serum of anaphylaxis patients, and their abundance correlates with the severity of the anaphylactic reaction.^9^ At the same time, these proteases play important roles in protection against invasive pathogens. For instance, mMCP-1-deficient mice show an increased susceptibility to *Trichinella spiralis* infection,^10^ whereas mMCP-6-deficient mice have higher mortality from *Klebsiella pneumoniae* infection.^11^ These observations indicate that MCs have multiple functions in host responses to allergens and infectious agents. MC-mediated immune responses can be both beneficial and harmful to our bodies. In this review, we summarize recent progress in our understanding of the molecular and cellular aspects of MC-mediated advantageous and detrimental immune responses.

## MCs as a beneficial and effective target for vaccine adjuvants

MC contains many granules rich in inflammatory mediators such as histamine and tumor necrosis factor-α (TNFα).^6^ These prestored mediators are immediately released upon MC stimulation that allows activated MCs to rapidly initiate immune responses to viral, bacterial or parasitic infections.^12^ For instance, MCs play important roles in the protection against parasitic (for example, helminthic) infections by orchestrating both early and late phases of immune responses and inducing lymph node hyperplasia at the beginning of infection.^12, 13^

**Figure 1:**
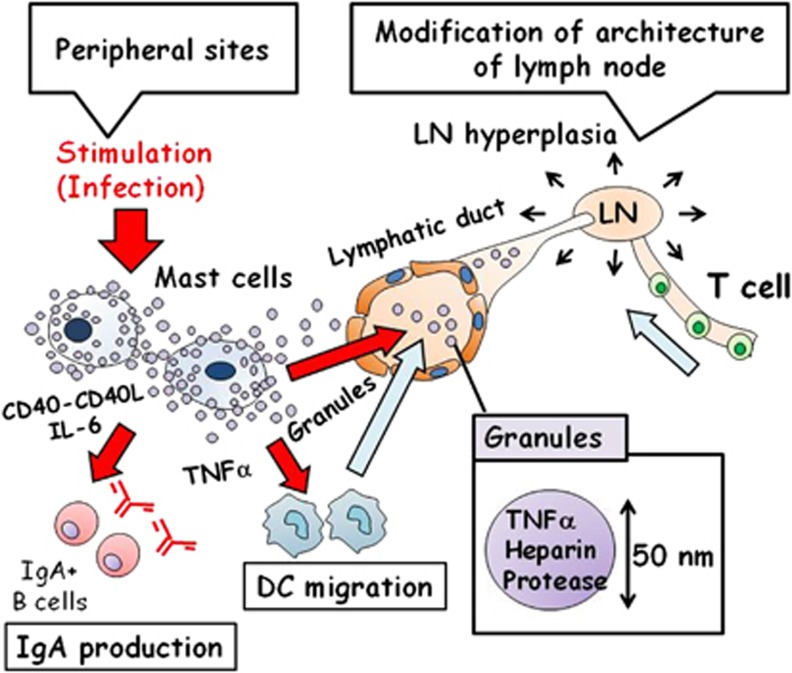
Activation of mast cells (MCs) and their role in immune cell orchestration. MC activation and degranulation are induced by various stimuli (for example, infection) at the peripheral sites. Interleukin 6 (IL-6) released by MCs and MC/B-cell interactions via the CD40/CD40L pathway enhance immunoglobulin A (IgA) production by B cells. Tumor necrosis factor-α (TNFα) released by MCs induces migration of dendritic cells (DCs) into the draining lymph nodes (LNs). MC-derived granules (∼50 nm diameter), containing TNFα and proteases, are delivered to the LNs and induce modification of their microarchitecture (for example, hyperplasia) that leads to accumulation of peripheral lymphocytes (for example, T cells) in the LNs.

Degranulated MCs release not only histamine and β-hexosaminidase but also insoluble granular particles composed of heparin proteoglycans and proteases; these granules are captured by phagocytic cells, such as DCs, and strengthen their ability to present antigens to the naive T cells.^14^ The granules are resistant to degradation and reach the lymph nodes, where they contribute to the rapid modification of lymph node microarchitecture, facilitating the T-cell/DC interaction (Figure 1).^12^ TNFα released from MCs orchestrates the recruitment of peripheral T cells and DCs into the draining lymph nodes during bacterial infections (such as *Escherichia coli* or *K. pneumoniae*), and effectively promotes antigen-specific immune responses (Figure 1).^12^ Recent observations have demonstrated that synthetic granules mimicking the MC-derived granules, composed of heparin–chitosan complexes with TNFα, can be used as a vaccine adjuvant (Figure 1).^15^ In mice, a synthetic granular adjuvant coadministered with influenza virus hemagglutinin effectively induces protective immunity against lethal influenza infection.^14^ MCs appear to possess at least two distinct granule subsets: VAMP-8 (vesicle-associated membrane protein 8)-dependent granules containing serotonin and cathepsin D, and VAMP-8-independent granules containing histamine and TNFα.^16^ Because TNFα released by MCs effectively induces an adaptive immune response, it is plausible that compounds inducing degranulation of only the VAMP-8-independent granules may serve as MC-targeting adjuvants.^16^

In addition to modifying the microarchitecture of lymph nodes to develop an adequate environment for the effective induction of antigen-specific immune responses, MCs can directly affect lymphocytes to accelerate the formation of the mucosal barrier. In mucosal compartments, IgA is important for protection against infection, and for cohabitation environment with commensal biota.^17^ Continuous production of optimal levels of mucosal IgA requires adequate stimulation by commensal bacteria and can promptly respond to pathogenic and invasive microorganisms. The importance of mucosal MCs in IgA production has been reported.^18^ Interleukin 6 (IL-6) secretion from MCs and the physical interaction between MCs and B cells via the CD40/CD40L pathway are the central mechanisms for the MC-dependent induction of mucosal IgA production by B cells (Figure 1).^18^ Intranasal administration of a vaccine (for example, against *Bacillus anthracis*) containing MC-activating compounds, such as compound 48/40 or cholera toxin A1 subunit DD/IgG immune complexes, induces antigen-specific IgA production in the nasal compartment and effectively suppresses *B. anthracis* infection in an MC-dependent manner.^19, 20, 21^ The mechanisms underlying the nasal IgA induction by MCs are not understood; however, activation of MCs located in the nasal-associated lymphoid tissue may be relevant.^20^

IL-1 cytokine family members, such as IL-18 and IL-33, are well-known MC activators produced by epithelial or myeloid cells. They enhance the induction of protective immunity (both T helper type 1 (Th1)- and Th2-type responses) against influenza infection when they are coadministered with vaccines; in MC-deficient mice, this protection is significantly reduced.^22^ In mice, IL-18 administration results in MC-mediated recruitment of DCs and T cells to the sites of inflammation.^23^ This suggests that IL-18 and IL-33 activate MCs to ensure effective development of antiviral immune responses.

In contrast to the protective roles of MCs in several viral infections, their activation leads to increased vascular permeability and pathological plasma leakage during hemorrhagic fever induced by Dengue virus.^24^ Therefore, in this case the suppression of MC activation is beneficial.^24^ RIG-I (retinoic acid-inducible gene 1) signaling in MCs mediates the inflammatory response during influenza A virus infection, and could potentially be an effective target to limit morbidity.^25^ Therefore, whereas targeting MCs by vaccine adjuvants should allow better protection from infectious diseases, excess activation of MCs should be avoided and/or controlled.

## MCs as effectors in allergic reactions in the gut

MCs are also known as effectors in allergic disorders.^26^ Accumulation of mucosal MCs located underneath the intestinal surface protects our bodies from parasites; however, once these MCs interact with nutrient-derived antigens, food allergies are induced.^26^ The importance of MCs for the development of food allergies has been shown in the murine model associated with allergic diarrhea.^27^ In this model, mice systemically sensitized with ovalbumin (OVA) and orally challenged with the same antigen develop allergic diarrhea, accompanied by the increased levels of intestinal MCs and antigen-specific IgE.^27^ In another model, mice presensitized with alum and OVA develop allergic diarrhea after oral OVA inoculation.^28^ Inhibitors of serotonin and histamine as well as depletion of MCs by an anti-c-kit monoclonal antibody reduce the occurrence of allergic diarrhea; therefore, MCs as well as serotonin and histamine derived from them are involved in the allergic diarrhea in this model.^28^ An interesting model of food allergy induced by the exposure to *Staphylococcus enterotoxin B* and an antigen (OVA) has been reported.^29^ In this model, exposure of *Staphylococcus enterotoxin B* to mucosal DCs enhanced expression of T-cell immunoglobulin domain and mucin domain (TIM)-4 and polarized TIM-1^+^ OVA-specific Th2 immune responses together with accumulation of MCs in the gut.^29^

As stated above, activation and accumulation of MCs at mucosal sites are important for the onset of allergic reactions in the gut, and are considered as an efficient target for treatment of such conditions.^27, 28, 29^ MCs are thought to spread from the bone marrow during the progenitor stage and to mature at the peripheral sites.^30^ The CCL2/CCR2 and leukotriene B4/BLT2 pathways have been reported as central for the MC progenitor migration to the lung, whereas the α4β7 integrin and CXCL2/CXCR2 pathways are thought to be involved in the gut.^30^ Commensal microbiota is required for the migration of colonic MCs, as the epithelial layer produces the CXCR2 ligand, such as CXCL2, in response to Toll-like receptor stimulation by commensal bacteria.^31^

Another factor affecting MC accumulation is sphingosine-1-phosphate (S1P) that plays an important role in food allergies.^32, 33^ Among five types of S1P receptors (S1P1–5), S1P1 is involved in MC migration.^34^ S1P production is increased during allergic reactions.^34^ S1P is produced via two sphingosine kinase isoforms, SphK1 and SphK2; SphK1 deficiency reduces both the circulatory S1P level and MC activation in anaphylaxis.^32^ The S1P1 modulator FTY720 reduces the number of MCs in the intestinal mucosa and prevents allergic diarrhea in mice.^33^ This indicates that reducing the accumulation of MCs in the intestinal mucosa could be therapeutically effective.

IL-9 is important for the expansion of MCs at the mucosal compartments. In IL-9-deficient mice, allergic symptoms, such as diarrhea in the food allergy model, are impaired, whereas mice overexpressing IL-9 develop intestinal mastocytosis, intestinal permeability and intravascular leakage.^35, 36^ These evidences indicated that to suppress allergic reactions in the gut, MC egress, migration/circulation, accumulation and proliferation at local tissues (the latter being stimulated by the cytokines produced by Th2 and Th9 cells, such as IL-3 and IL-9) should be inhibited.^36^

## MCs sense endogenous ligands and promote mucosal inflammatory disorders

MCs also play important roles in the maintenance of mucosal homeostasis in the gut, including the intestinal barrier function. MC-deficient or chymase-deficient mice show decreased epithelial turnover and permeability; the rate of epithelial cell migration up the villus–crypt axis in the jejunum of these mice is reduced by ∼20%, resulting in altered villus morphology.^37^ Optimal epithelial turnover is maintained by MC-derived chymase, partly by regulating claudin-3 expression.

Although MCs are important for the healthy intestinal epithelium,^37^ aberrant MC activation leads to inflammation. The numbers of activated or degranulated MCs are greater in colon specimens from Crohn's disease patients than in normal colon tissue specimens.^38^ In patients with inflammatory bowel diseases (IBD), local production and circulation of tryptase, released from MCs in the gut, is increased.^39^ An elevated level of tryptase has also been detected in tissue specimens from patients with IBD.^39^ MC-derived tryptase induces acute intestinal inflammatory responses by inducing a variety of mediators, such as IL-1β, IL-6 and matrix metalloproteinases MMP3 and MMP13.^39^ Recent studies by us and others indicate that MC-derived proteases (for example, tryptase) and inflammatory cytokines (for example, TNFα) are involved in the progression of inflammatory symptoms in the gut.^39, 40^

**Figure 2:**
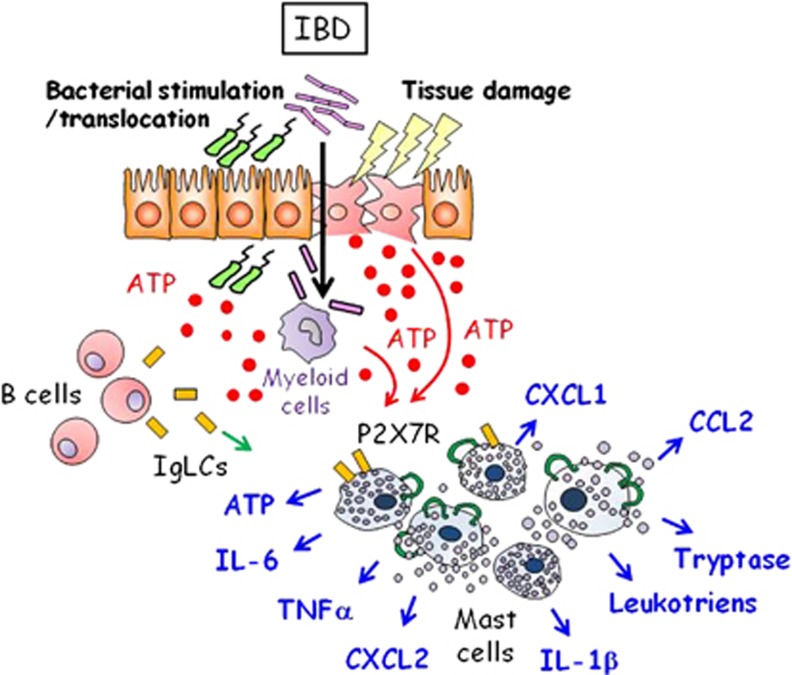
Activation of mast cells (MCs) in intestinal inflammation. Colonic MCs are activated by Ig-free light chains (IgLCs) produced by B cells and by adenosine triphosphate (ATP) released by damaged epithelial cells, activated monocytes by bacterial stimulation and bacteria. Extracellular ATP is recognized by the P2X receptors on MCs (green loops) and leads to production of inflammatory cytokines (for example, interleukin (IL)-1β, IL-6 and tumor necrosis factor-α (TNFα), chemokines (for example, CCL2, CXCL1 and CXCL2), lipid mediators (for example, leukotrienes) and proteases (for example, tryptase).

In the inflamed tissues, MCs are degranulated, and therefore it is important to elucidate the molecular mechanisms of MC activation. Ig-free light chains (IgLCs), which had been considered as by-products of immunoglobulin production by B cells, are involved in various inflammatory disorders.^41^ Increased serum concentrations of Ig-free light chains and their presence in colon specimens from IBD patients have been reported.^41^ Ig-free light chains bind to MCs (Figure 2) and increase vascular permeability in the colon in a mouse model of IBD.^41^ Yet, MC activation is also observed in B cell-deficient mice; therefore, we have suggested the existence of multiple MC activators during intestinal inflammation.^40^

Extracellular adenosine triphosphate (ATP) is considered as one of the danger-associated molecular patterns.^42, 43^ ATP is released from necrotic cells, commensal bacteria and activated monocytes.^42, 43^ MCs may also release or regenerate ATP to the extracellular compartments.^40^ ATP levels are increased in the peritoneal cavity of mice with graft-versus-host disease.^44^ ATP release has been reported to be significantly higher in colorectal biopsies from mice with colitis than in those from control mice.^40^ Extracellular ATP concentration is tightly regulated *in vivo* to maintain immune homeostasis.^42, 43^ In mice lacking the ectonucleotidase CD39, which dephosphorylates extracellular ATP, intestinal inflammation in experimental colitis is exacerbated.^45^ These observations indicate the importance of extracellular ATP in intestinal inflammation.

Extracellular ATP induces a wide range of pathophysiological responses via activation of purinergic P2 receptors at the cell surface.^42, 43^ P2X purinoceptors (P2X1–7) act as ATP-gated ion channels.^42^ P2X7 is involved in various inflammatory conditions, such as asthma, contact hypersensitivity and graft-versus-host diseases.^44, 46, 47^ In the colon tissue, MCs express high levels of P2X7.^40^ Our and other previous studies indicate that extracellular ATP stimulates MCs to release inflammatory cytokines (for example, IL-1β, IL-6 and TNFα), chemokines (for example, CCL2 and CXCL2) and lipid mediators (for example, leukotriene B4) in a P2X7-dependent manner (Figure 2);^40, 48^ these compounds play a critical role in the MC-mediated intestinal mucosal inflammation. Furthermore, P2X7-expressing MCs accumulate at the inflammatory sites in the colons of Crohn's disease patients.^40^

MC activation is essential for the basal level of mucosal homeostasis, including healthy turnover of intestinal epithelium, whereas excessive activation induces intestinal inflammation.^37, 40^ Therefore, the endogenous and exogenous factors responsible for MC activations need to be carefully examined before they can be considered as effective targets for the prevention and treatment of IBD. Direct MC interactions with other immune cell populations, such as T cells, DCs and innate lymphoid cells at the peripheral sites, are important for the development of inflammation.^6, 49^ Interactions with fibroblasts are also necessary for the MC development.^50^ These cell–cell interactions and communications need to be further understood to enable treatment of MC-dependent inflammatory diseases.

MCs may also induce immune suppression or tolerance; IL-2 or IL-10 produced by MCs suppresses skin inflammation.^5^ In radiation proctitis, MC-derived mediators such as chymase, tryptase and histamine are involved in the development of a proinflammatory phenotype of the muscularis propria smooth muscle cells and the regulation of acute tissue neutrophil influx.^51^ These regulatory-type MCs may be produced in tissue- and environment-dependent manner.^52^ The precise mechanisms of acquiring tissue-specific or disease-specific MC phenotypes need to be elucidated, as dysregulation of tissue-specific regulatory mechanisms in MCs may cause tissue-specific inflammatory disorders.

## Concluding remarks

Accumulated knowledge indicates that MCs have unique and diverse functions; they play protective and regulatory roles, and are also involved in inflammation. A better understanding of the spatiotemporal dynamics of MCs during homeostatic and disease conditions including infection and acute/chronic inflammation is needed. Although various types of MC-deficient mice with mutated c-kit, mast cell proteases or IL-4 enhancer region (MaS-TRECK) have recently been generated and analyzed,^53^ we need to be aware of the difference in the function and development of MCs between mice and humans. Further investigations using MC-deficient mice and human MCs will elucidate the diversity of MC functions, and help to develop strategies will be horizon to control and regulate MCs to maintain homeostasis, and to prevent or treat diseases.
